# Hepatitis C and Thalassemia: A Story with (Almost) a Happy Ending

**DOI:** 10.3390/pathogens12050683

**Published:** 2023-05-05

**Authors:** Raffaella Origa

**Affiliations:** 1 Ospedale Pediatrico Microcitemico, Via Jenner Sn, 09121 Cagliari, Italy; raffaella.origa@unica.it; 2 Department of Medical Sciences and Public Health, University of Cagliari, Cittadella Universitaria di Monserrato Strada Provinciale 8, 09042 Cagliari, Italy

**Keywords:** transfusion, HCV, thalassemia, hepatocellular carcinoma, liver transplantation

## Abstract

Donor screening has nearly eliminated the risk of hepatitis C virus post-transfusion transmission in resource-rich settings. Moreover, the use of direct antiviral agents made it possible to treat the majority of patients with thalassemia and hepatitis C. However, this achievement, while extremely significant, does not erase the effects of the virus in terms of fibrogenesis and mutagenic risk, and adult patients with thalassemia are facing the long-term consequences of the chronic infection both on the liver and extrahepatically. As in the general population, it is in mainly patients with cirrhosis who are increasing in age, even though they are now HCV RNA-negative, who are at risk of hepatocellular carcinoma, which continues to be statistically much more frequent in individuals with than without thalassemia. In certain resource-limited settings, the World Health Organization has estimated that up to 25 percent of blood donations do not undergo screening. It is therefore not surprising that hepatitis virus infection is still the most prevalent in patients with thalassemia worldwide.

## 1. Introduction

In 2022, the World Health Organization (WHO) estimated that approximately 58 million people had chronic hepatitis C virus (HCV) infection [1]. Although this number represents a decrease from 2015, which was the first year of commercial release of direct antiviral agents (DAAs), at least 1.5 million new infections occur each year, and forecasts suggest we are not currently on track in order to achieve global elimination targets by 2030 [2].

In the early 1990s, immediately after the HCV screening test became available, the prevalence of chronic HCV infection among patients with thalassemia ranged from 4% in Turkey to 85% in Italy [3,4]. In the latter, a prevalence of clinically significant fibrosis of 45% and a risk of contracting post-transfusional non-A, non-B hepatitis of about 20 cases per 1000 units of blood were observed. HCV antibodies were also reported in 23% of patients in the United Kingdom, and 35% in the United States [5,6].

Donor screening has nearly eliminated the risk of post-transfusion infection in resource-rich settings. Moreover, the availability of safe and effective drugs has substantially eradicated HCV in the multitransfused patients. However, in certain resource-limited settings, the WHO has estimated that up to 25 percent of blood donations do not undergo screening [7]. It is therefore not surprising that HCV infection is still the most prevalent in patients with thalassemia worldwide [8]. Thus, the hepatitis C in patients with hemoglobinopathy who need transfusions has two sides: in Western countries, the risk of new cases is minimal, but the adult patients are facing the long-term consequences of the past chronic infection both on the liver and extrahepatically; in many world regions, it is mainly children who continue to become infected by transfusion.

## 2. Methods

A comprehensive literature search of peer-reviewed articles published from inception to March 2023 was conducted to identify relevant studies, using the electronic databases MEDLINE/Pubmed, Scopus, Embase, Web of Science, and the Cochrane Library. The search included title, abstract, keywords, and medical subject headings (MeSH) related to hepatitis C in thalassemia, hepatocellular carcinoma and thalassemia, antiviral treatments in thalassemia, and liver transplantation in thalassemia. The references in the fetched articles were also reviewed to find other studies that may not have been identified in the primary search.

## 3. Natural History of Hepatitis C in Thalassemia

It is generally stated that in the general population, HCV infection is self-limiting in 15–40% of patients, while 75–60% of infected patients do not clear the virus within 6 months [9]. A review of 675 individuals showed that clearance of infection ranged from 0% to 80% with a weighted mean of 26% [10]. This considerable variance depends on the fact that the population studies to determine the rate of persistence are few and may be biased by the mode of ascertainment. They frequently involve the prospective study of symptomatic individuals, who are more likely to clear the virus [11,12]. The chronicity rate appears to be lower in younger individuals [13]. In this respect, long-term follow-up studies show that 55% to 60% of children with post-transfusion hepatitis after heart surgery and 59.9% of hemophiliacs infected with HCV before the age of two years are still HCV RNA-positive as adults [14,15]. A similar proportion (55.8%) was found in a large cohort of people with thalassemia in Sardinia, which suggests that liver iron overload does not play a significant role in determining chronicity. Nine of the children (17.3%) who had been previously infected and had cleared the virus had re-infection with HCV [16]. In an Egyptian study, patients with thalassemia and recent HCV infection were followed for spontaneous resolution of acute HCV or development of chronic hepatitis [17]. Patients with acute HCV without thalassemia were also enrolled in the study. Other than the different iron profile, the two groups did not have differences in demographics or clinical features. Spontaneous resolution occurred in 10 of the 57 patients (17.5%) with coexisting acute HCV and thalassemia (28.5 ± 9.2 years), compared to 24 of the 69 patients without thalassemia (34.78%) (*p* = 0.043). Although the difference was statistically significant, it is not possible to draw definitive conclusions: the number of patients considered is limited and, more in general, the cohorts are non-homogeneous and there are differences in enrollment criteria in the few studies on subjects with thalassemia, as the same authors of the Egyptian study claim.

A large body of data suggests that the natural history and treatment response of HCV positive patients is largely determined by complex host–virus interactions. Whereas no study in subjects with hemoglobinopathies has demonstrated a significant association between viral factors and spontaneous HCV clearance as already shown in the general population, the genetic variants in IL-28B gene linked with the spontaneous clearance of HCV genotype even in the patients with thalassemia [16,18,19,20]. 

HCV genotypes reported in patients with thalassemia closely track the genotypic distribution in the countries in which they receive transfusion therapy [21]. For example, regarding Europe, genotype 1 is the most frequent (between 50 and 70%), and similar frequencies were reported in Italian thalassemia patients [16,22,23].

Angelucci et al. (2002) found that HCV infection and iron overload were independent factors in the progression of fibrosis in thalassemia patients following bone marrow transplant [24]. Other authors report that in patients with thalassemia and HCV-related chronic hepatitis, fibrogenesis has a more striking relationship with liver siderosis than with viral load [4,16]. In this regard, Lai et al. (2013) showed that among sex, time interval between acute hepatitis and liver biopsy, HCV RNA status, and liver iron according to Sciot semiquantitative iron scoring (scores ranging from 0 to 4), only the last was significantly associated with liver fibrosis (*p* = 0.048), when analyzing 32 liver biopsies from transfusion-dependent thalassemia patients (13 HCV RNA-negative and 19 HCV RNA-positive) [16]. In the same article, increased transaminases were registered in almost half of HCV RNA-negative patients. On the other hand, it is well known that hepatocellular carcinoma may occur both in HCV-positive and HCV-negative patients with thalassemia [25]. While the progression of fibrosis was similar in patients with thalassemia compared with the general population in the study by Lai et al. (2013), other authors report an accelerated hepatic fibrosis [16,17,26,27]. This contradiction is probably merely apparent because it is ascribable to the cumulative effect of HCV-associated liver damage and increased iron overload, particularly in patients with inadequate chelation. This may suggest that iron chelation therapy may reduce the additive or synergistic effect of HCV and iron on the rate of progression of liver fibrosis, even though it may not be able to completely remove iron from the liver [15,26].

## 4. Hepatocellular Carcinoma

After the introduction of regular transfusions and until the 2000s, liver disease ranked after heart disease and infection as a cause of death among adolescents and adults with thalassemia [28,29,30]. Since the early 2000s, however, the dramatic reduction in cardiac iron overload-related mortality as well as the consequent increased survival resulted in the hepatic causes of death overtaking those of cardiac causes [31,32,33]. The hepatic cause of death included non-neoplastic complications from HCV (cirrhosis, liver failure) and hepatocellular carcinoma.

Currently, cancers are the second leading cause of death in the ageing thalassemia patient, and hepatocellular carcinoma is the only cancer that has been unequivocally shown to be more frequent in people with thalassemia than in the general population [34].

The first case of hepatocellular carcinoma in thalassemia reported in the literature in 1986 was a 22-year-old man who had received subcutaneous deferoxamine chelation for less than 6 years. HCV was not identified at that time [35]. In 2004, the first Italian retrospective study on the incidence of hepatocellular carcinoma collected 22 additional cases in patients with hemoglobinopathies, 11 of which had thalassemia intermedia, and 8 thalassemia major. A total of 86% of them were anti-HCV positive and 77% HCV RNA positive [36]. In the 2014 update, 60 new cases of hepatocellular carcinoma were reported to the Italian registry out of 5855 thalassemia patients (52% with thalassemia major and 45% with thalassemia intermedia). Two patients had hemoglobin S/thalassemia. Only four patients, all affected by thalassemia intermedia, did not have previous signs of HCV infection [37]. These data, together with those from sporadic case reports from other publications on Italian, Greek, Lebanese, and Iranian patients indicates that, in thalassemia, two groups of patients are particularly at risk of developing hepatocellular carcinoma [38,39,40,41]. The first includes patients with the phenotype previously referred to as thalassemia major, for whom active HCV infection is found in the majority of cases; the second comprises patients with the phenotype previously referred to as thalassemia intermedia—whether transfused or not but usually inadequately chelated—for whom the factor with the greatest impact in influencing the development of hepatocellular carcinoma is iron. Also noteworthy, the above studies show an increased risk of developing hepatocellular carcinoma in patients with thalassemia intermedia, because of the worse treated iron accumulation and the improved survival compared with those who have thalassemia major. The carcinogenicity of iron is related to the generation of reactive oxygen species leading to genotoxicity, the acceleration of fibrosis to cirrhosis through the activation of stellate cells and the profibrogenic effects of lipid peroxidation. In addition, iron leads to immunological dysregulation, which weakens immune surveillance against cancer [25,42,43,44,45,46]. First, tumor cells exhibit iron addiction and iron has a specific role in macrophage polarization, T-cell activation and differentiation and B-cells antibody response. Second, tumor cells divert immune cells resident in the tumor microenvironment to satisfy their enhanced demand of iron supply. In turn, the microenvironment enriched in iron released from inflammatory cells supports cancer cell progression by inducing lymphocytes death due to oxidative stress and by reducing the capacity of antigen-presenting cells to prompt an effective anti-tumor immune response.

The presence of the two risk factors explains why thalassemia can be considered an independent factor for hepatocellular carcinoma, and the age of onset is significantly lower than in the general population with most patients developing the tumor at age <50 years. An independent role of hepatitis B virus in liver carcinogenesis in patients with thalassemia has not been demonstrated, although a minority of patients with thalassemia and hepatocellular carcinoma were also HBsAg-positive. This may be partly due to the fact that most reports come from developed countries or countries with low HBV incidence due to effective risk factor control, HBV vaccination, and treatment. Usually, transfusion-related hepatitis C is a key factor in liver damage among patients with thalassemia through the increased risk of cirrhosis. However, in this setting, it has been observed that hepatocellular carcinoma can also develop in the cirrhosis-free liver. This suggests that HCV infection might be an independent risk factor for hepatocellular carcinoma in patients with thalassemia, even in the absence of cirrhosis [47,48]. This was reported for the first time in an HCV-infected female with thalassemia intermedia in the presence of iron overload [47]. Indicating the exact proportion of patients with thalassemia who develop hepatocellular carcinoma in the absence of cirrhosis is currently impossible because these data are often unavailable at the time of diagnosis.

The most recent evidence about hepatocellular carcinoma in beta thalassemia confirms the previous data, especially regarding the complex relationship between iron, HCV infection, and hepatocellular carcinoma as well as the possibility of tumor development in the absence of cirrhosis [48].

The eradication of HCV may improve the quality of life of the patients with thalassemia, but the advantage in terms of decrease of liver complications, hepatocellular carcinoma, and overall survival requires more data and longer observation. In a recent report from Greece, 42 transfusion-dependent thalassemia patients with hepatocellular carcinoma have been included. Most of them (78.5%) were anti-HCV positive, and 59.5% had been successfully treated with either Peg-Interferon alpha ± ribavirin or with the new direct antivirals. Underlying cirrhosis was present in 78.5% of patients [49].

Similar findings were highlighted by Ricchi et al. (2021) and by Origa et al. (2023) in the largest population with thalassemia and hepatocellular carcinoma described so far (80 cases of hepatocellular carcinoma in 4631 Italian patients with hemoglobinopathies) [34,35,36,37,38,39,40,41,42,43,44,45,46,47,48,49,50]. In the latter study, the risk of developing hepatic cancer was significantly higher (*p* < 0.001) in subjects with transfusion-dependent thalassemia in comparison with the other hemoglobinopathies. Furthermore, male sex, positive HCV antibodies, and HCV RNA detection were significant risk factors for hepatocellular carcinoma on univariate analysis. All of these, with the exception of anti-HCV positivity, were confirmed to be significant by multivariate analysis. Fourteen patients (17.9%; ten with transfusion-dependent thalassemia, three with non-transfusion-dependent thalassemia, and one with sickle cell disease) were negative for anti-HCV. In all those with known data, there was a history of elevated liver iron concentration and/or serum ferritin. Their median age at diagnosis was 55.1 years, and only one patient was under 45 years. They were older than those who progressed to hepatocellular carcinoma and were anti-HCV-positive and HCV RNA-negative (median age, 47.3 years; IQR, 52.7–68 years; *p* = 0.021) or HCV RNA-positive (median age, 45.3 years; IQR, 50.9–78.8 years; *p* = 0.011). A total of 19 patients who were negative for HCV RNA at diagnosis (13 of 18 with cirrhosis; information not available for one patient) were previously treated with antivirals and achieved sustained virological response (SVR). In 11 of these patients, viral clearance was achieved with DAAs. However, antiviral therapy, particularly DAA, was protective against developing hepatocellular carcinoma (*p* < 0.001). As demonstrated in those without thalassemia, in this group of patients, the presence of cirrhosis at the time of antiviral therapy appears to be the most impactful factor in the residual risk of liver tumor [34,50,51,52]. In patients in whom a history of cirrhosis was not known, it was also possible to identify a history of relevant hepatic iron accumulation. Table 1 collects patients with beta thalassemia who developed hepatocellular carcinoma after reaching SVR published to date.

In the general population, close monitoring with liver imaging for the development of hepatocellular carcinoma after SVR is recommended twice a year indefinitely, at least in patients with HCV cirrhosis [52]. This is even more applicable in patients with thalassemia where other risk factors persist, the most relevant being obviously liver iron overload. According to Moukhadder et al. (2017), the patients with thalassemia should undergo liver ultrasound twice a year in case of cirrhosis, HCV and/or hepatitis B virus infections, liver iron concentration ≥ 5 mg/g dw (non-transfusion dependent patients), or ≥7 mg/g dw with or without serum ferritin values ≥ 1000 ng/mL (transfusion-dependent patients) [25]. Origa et al. (2023) recommended expanding the existing indications for biannual ultrasound to include patients aged 45 years or older with β-thalassemia and past (or present) significant hepatic iron accumulation, even in the absence of a history of chronic HCV [34]. However, it is not possible to establish a hepatic iron accumulation cut-off below which there is no risk of hepatocellular carcinoma, and the risk depends not only on the degree of accumulation but also on the duration of exposure. Consequently, it is probably more correct to recommend biannual ultrasound scanning for all thalassemia patients who have reached 40 years of age, whether they are anti-HCV-positive or not.

Finally, there are no data to suggest that the management of hepatocellular carcinoma should differ in patients with or without beta-thalassemia. A multidisciplinary approach involving hepatologists, radiologists, and oncologists together with the expert in thalassemia is essential in identifying a tailored treatment. The adoption of the same therapies currently in use, either loco-regional, or resective, or chemotherapeutic, or palliative have led to a survival improvement in beta-thalassemia patients with hepatocellular carcinoma from 2004 (3.5 months median time from diagnosis to death) to 2014 (median of 11.5 months) [36,37]. The median survival time after hepatocellular carcinoma reported by Origa et al. (2023) was 4 years, and the 5-year survival rate was 39.5% [34]. This value is significantly higher than that shown in the general population—approximately 20% in Italy, 30% according to Bannon et al. (2019) [54,55]—and suggests that what may make a difference is not only increased treatment options but also regular screening and early diagnosis.

The role of liver transplantation in patients with hemoglobinopathies remains unclear. Probably, given the increased life expectancy and improved health status of these patients, liver transplantation is a therapeutic option still too infrequently considered [56,57]. Three HCV RNA-positive patients who underwent liver transplantation were described by Borgna Pignatti et al. (2014), two of whom were dead at the time of the report, one of cirrhotic liver failure and the other of meningococcal sepsis [37]. Seven other patients were described by Origa et al. (2023) [34]. Five were still alive at the last follow-up (6.4 years after liver transplantation as a median, min-max 0.1–17.2). The remaining two patients died because of liver failure and sepsis, 1.9 years and 8 months after transplantation, respectively.

## 5. Antiviral Treatments

Treatment of chronic HCV in thalassemia historically began with interferon alpha monotherapy. The variable response rates observed with this treatment were considered due to the coexistence of liver iron overload, often associated with more advanced liver disease, by part of the authors [58,59,60,61,62]. The absence of cirrhosis, low liver iron content, and infection with non-1b HCV type were independently associated to complete sustained response upon multivariable analysis [63]. Moreover, the wide array of adverse effects of interferon alpha including the flu-like syndrome and fatigue, hemolytic anemia, cytopenia, and neuropsychiatric side effects became problematic in a significant proportion of patients. The same adverse events could occur with the combination of pegylated interferon alpha and ribavirin, which was the recommended treatment until a few years ago and was not well tolerated by thalassemia patients, leading to both low compliance and SVR rates. In addition to side effects from interferon alpha, in fact, ribavirin-associated hemolysis can determine an increase in the need for transfusions and the consequent worsening of iron overload, leading in turn to an eventual progression of liver damage [64,65]. IFNL3 polymorphisms have been demonstrated to influence the stage of fibrosis and spontaneous or interferon-induced viral clearance [20,22]. The role of iron overload in conditioning SVR in thalassemia patients treated with pegylated interferon alpha has been much debated [66,67]. Although an international panel recommended that intensification of chelation should be considered before starting antiviral treatment in patients with severe iron overload, they emphasized that there was little evidence of its benefit in thalassemia patients [64].

In the early 2010s, new targets in HCV therapy DAAs were developed to different HCV non-structural (NS) proteins such as NS3 protease, NS5A replication-associated protein, and NS5B polymerase. First-generation protease inhibitors, used in combination with pegylated interferon alpha and ribavirin to treat genotype 1 infection, were not tested in thalassemia patients because of their significant adverse effects. Finally, the majority of patients infected with HCV who did not spontaneously clear the virus were still HCV-RNA-positive when DAAs became available. As in subjects with chronic hepatitis C without thalassemia, even in those with thalassemia, DAAs therapy has been revolutionary. The majority of patients experienced no side effects during treatment. Furthermore, the side effects reported were negligible compared to those seen with previous antiviral therapies, including interferon alpha. Even in the subgroup of patients treated with ribavirin, the new antiviral treatments had little effect on blood consumption because of their reversibility and short duration. Moreover, the probability of SVR has also dramatically increased: it was between 28 and 66% in patients under interferon alpha monotherapy and between 31 and 93% in the largest cohorts of patients treated with the combination pegylated interferon alpha and ribavirin compared to percentages close to 100% in patients treated with DAAs. These brilliant results have been possible also thanks to the development in recent years of highly effective and increasingly selective drugs toward different viral genotypes [68,69,70,71]. No significant differences in terms of SVR between patients with or without cirrhosis were detected. In an Italian cohort of patients with hemoglobinopathies, chronic HCV infection, and advanced liver fibrosis, 139 patients received DAAs. They completed 12 weeks of post-treatment follow-up. The SVR 12 weeks after the end of treatment (93.5%) was comparable with that typically observed in cirrhotic patients without hemoglobinopathies [69]. In Sardinia, a total of 99 patients, including 26 diagnosed with cirrhosis, were treated with at least one dose of different regimens of DAAs. The regimens were safe and well tolerated. Two of the patients died during treatment after becoming HCV RNA-negative, while another patient voluntarily discontinued treatment. Final SVR in patients who completed treatment was 100%, with 97% (96/99) in the intention-to-treat analysis [70]. Another large cohort of Italian thalassemia patients HCV genotype 1 or 4 including naïve with cirrhosis (16%) and prior treatment failure without cirrhosis received a combination of sofosbuvir and ledipasvir for 12 weeks (without ribavirin). SVR was reported in 98% of patients (95% CI 95.3%-100%). Cirrhotic and previous treatment failure achieved 100% SVR. Adverse events including fatigue, headache, nausea, decrease in hemoglobin or increase in ferritin levels were rare and not common [69]. Similar efficacy and lack of specific adverse events has been demonstrated Irrespectively of the different iron chelators in use [69,70,71]. Experiences with DAAs in patients with thalassemia have been reported worldwide [72,73]. Commonly, the patients treated in resource-limited countries are children or adolescents with results comparable to the adults [74,75,76]. Given the excellent global results of the DAAs, with outcome equally positive in patients with no iron and with moderate or severe liver iron overload, the role of hepatic siderosis in conditioning SVR seems extremely unlikely.

## 6. HCV and Extrahepatic Manifestations in Patients with Beta Thalassemia

The hepatitis C virus has been shown to affect many tissues other than the liver. However, of the many extrahepatic manifestations that have been associated with HCV, only a few have been shown to be directly related to HCV infection of extrahepatic tissues while HCV-triggered immune-mediated mechanisms account for most of the others. It is estimated that up to 74% of patients with chronic hepatitis C can develop at least one extrahepatic manifestation [77].

**Figure 1:**
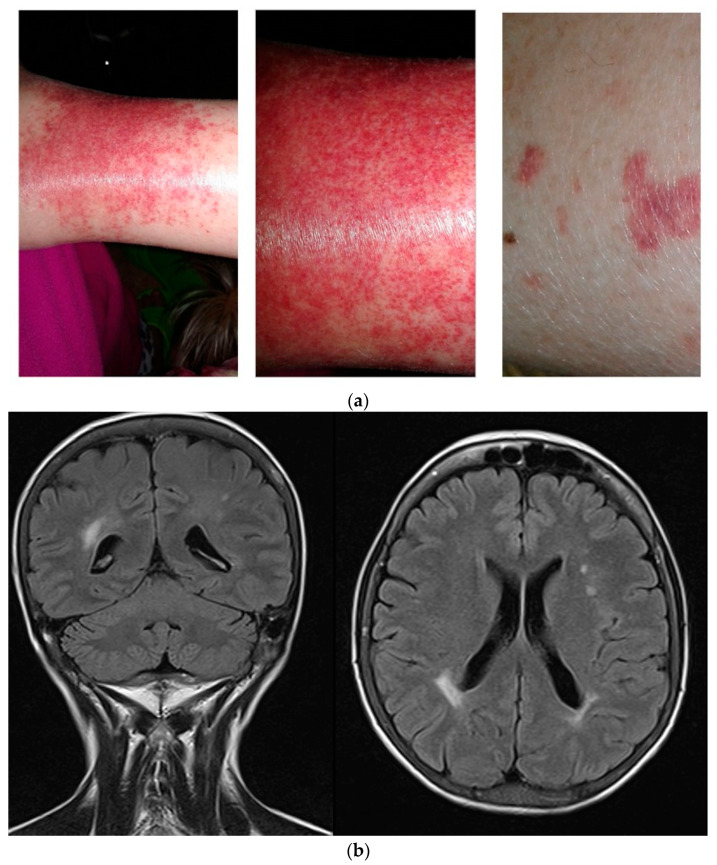
Ankle cryoglobulinemic purpura (**a**) and signs of cerebral vasculitis in the coronal and axial plane at magnetic resonance scan with contrast (**b**) in a 44-year-old woman with thalassemia who had been infected by HCV in her infancy. The diagnosis of cryoglobulinemic purpura was certain in 2009 when the patient, after repeated episodes of severe bilateral ankle pain and purpura, underwent skin biopsy. The magnetic resonance scan was performed because of a severe headache, and the cerebral vasculitis can be considered an occasional finding. The patient achieved SVR in 2016 with DAAs while cryoglobulins became negative some months later. Despite HCV RNA and cryoglobulin negativity, the woman still suffers from a sensorimotor neuropathy with moderate gait instability and repeated falls.

The virus replication in peripheral blood mononuclear cells may be etiologically implicated in B-cell clonal proliferative disorders such as mixed cryoglobulinemia and non-Hodgkin’s lymphoma [78]. HCV infection is considered to be the main cause of mixed cryoglobulinemia, especially type II. Various studies have reported between 10% and 60% of HCV-infected patients with cryoglobulinemia, which is nearly always asymptomatic [79,80]. In thalassemia, cryoglobulinemia-associated symptoms such as asthenia, arthritis, and proteinuria are very common and may have a variety of causes, making them difficult to attribute to cryoglobulinemia. In 1995, a study to determine the incidence of cryoglobulinemia and associated symptoms in 264 HCV-positive transfusion-dependent thalassemia patients from Sardinia found the presence of cryoglobulins in 25.8% of cases [81]. A high prevalence of cryoglobulinemia (28.6%) in that same population was confirmed many years later [70]. Only one patient presented with symptoms clearly attributable to the disease: repeated episodes of lower limb purpura with severe arthralgia and painful peripheral neuropathy confined to the lower limbs with predominant sensory impairment (Figure 1a) and a magnetic resonance imaging suggestive of cerebral vasculitis (Figure 1b).

Cryoglobulinemia was reported in 35 (66.0%) patients with chronic HCV infection by Perniola et al. (1999) experiencing cutaneous lesions (purpura, Raynaud’s phenomenon, nodules, and leg rash), peripheral neuropathy, and sicca syndrome symptoms [82]. Musculoskeletal symptoms (bone pain, arthralgia, and myalgia), weakness, splenomegaly, lymphadenopathy, skin ulcers, and proteinuria were more common than in HCV-RNA-positive patients without cryoglobulins. However, the difference did not reach statistical significance, possibly because of the partial overlap between cryoglobulinemia and beta-thalassemia. When treated with DAAs, patients with HCV-related mixed cryoglobulinemia achieve high rates of viral clearance, which is often associated with improvements in clinical symptoms and immunological profiles [83]. However, it is possible that cryoglobulinemia has resulted in irreversible complications that are destined to last and show lifelong signs such as peripheral neuropathy and cerebral vasculopathy [70,84]. Furthermore, as reported in the general population, not all patients exhibit negative cryoglobulins at SVR, if treated with DAAs [61,71,73]. In cryoglobulinemic vasculitis, B-cell proliferation may eventually reach an HCV-independent autonomous phase [85]. Long-term follow-up of patients with thalassemia major and mixed cryoglobulinemia treated with DAAs is therefore important, with periodic evaluation of lymphocyte subpopulation analysis, even beyond the achievement of cryocrit negativity [70].

Four patients developing non-Hodgkin’s lymphoma before the introduction of DAAs were reported by Ponti et al. (2019) [70]. All four were HCV RNA-positive and did not have mixed cryoglobulinemia. The correlation between HCV and hematological neoplasms in thalassemia has neither been demonstrated nor studied. It is even controversial whether there is an elevated risk of hematological neoplasms and lymphomas in particular in this category of patients. An increased risk has been highlighted by Chung et al. (2015) but recently disconfirmed by Origa et al. (2023) [34,86].

Recent studies suggest an association between HCV infection and a higher incidence of major adverse cardiovascular events such as coronary heart disease, heart failure, stroke, and peripheral artery disease in comparison to the general population [87]. Moreover, cardiac arrhythmias such as long QT interval, bradyarrhythmia, tachyarrhythmia, atrial fibrillation, and sick sinus syndrome are also more common among HCV-infected patients as well as cardiomyopathies and valvular heart diseases [88,89,90,91,92,93]. Although cirrhosis and hepatitis C in general are considered systemic diseases and a correlation between HCV infection and the risk of heart failure in thalassemia patients has been reported by some authors [65], the impact that chronic hepatitis C has had on the hearts of people with thalassemia is, on the other hand, difficult to quantify. Indeed, iron acts as a confounding factor, and the same proportionality that existed in the past between number of transfusions and the risk of contracting hepatitis C was also there with the risk of iron-related heart disease. This may cause heart disease and hepatitis C to coexist in many of the studies that have examined the prevalence of complications in patients with thalassemia. In the general population, HCV treatment has been reported to decrease the incidence of atherosclerotic heart diseases and arrhythmia, either using old interferon alpha-containing regimens or DAAs agents [94,95,96,97,98]. Nevertheless, these data are lacking regarding subjects with thalassemia. The attempt to demonstrate the effect of DAAs on contractile function by Ponti et al. (2019) was unsuccessful because the majority of the cirrhotic patients under study had a normal left ventricular ejection fraction at the start of the treatment [70].

The prevalence of diabetes in thalassemia has been shown to correlate with HCV infection, as well as with pancreatic and cardiac iron, and serum ferritin [99,100]. In the general population, compared with healthy controls and patients with other liver diseases, patients with chronic hepatitis C have a threefold increased risk of developing type II diabetes [101,102]. The development of cirrhosis further increases the risk (19.6% to 50%) since the associated liver insufficiency also inhibits glucose metabolism [103]. Viral effects on insulin resistance (both intrahepatic and extrahepatic) and altered glucose metabolism associated with cirrhosis or fibrosis secondary to HCV infection are the most important factors underlying the increased risk of diabetes in hepatitis C [103]. Other mechanisms include pro-inflammatory cytokines, chemokines, and other immune-mediated mechanisms. Finally, although the exact cellular and molecular mechanisms behind this association are not completely understood, chronic HCV infection might increase the risk of osteoporosis in patients with thalassemia as already shown in the general population [104,105]. In this regard, according to Meloni et al. (2023), HCV represents an independent risk factor for low bone mineral density and osteocalcin may play a role in the loss of bone mass associated with HCV infection [106].

## 7. Residual Risk of Hepatitis C in Children and Adults with Thalassemia

Progressive implementation of nucleic acid-amplification technology (NAT) screening for HIV, HCV, and HBV has reduced the residual risk of infectious-window-period donations, such that per unit risks are <1 in 1,000,000 in the United States and other high-income countries [107]. The high level of transfusion safety in high-income countries has not been matched in most low-to-middle-income countries with challenges spanning the entire blood-safety chain from donor selection to post-transfusion surveillance [108,109,110,111]. In India, Biswas et al. (2022) reported transfusion-transmitted infections in 25 out of 328 children with transfusion-dependent thalassemia, and hepatitis C was the most common (34.5%) with a significant negative impact on their quality of life [112]. Even higher prevalence of anti-HCV antibodies was found in other studies in the same part of the world [113]. The implementation of NAT in Egypt is limited due to the financial burden. This country has the highest recorded prevalence of HCV antibodies in the world and a high prevalence (8.9%) of HCV carriers among blood donors (8.5%) [114,115]. It is therefore not surprising that a high prevalence of HCV infection is reported among Egyptian children with thalassemia, indicating that HCV remains a major health challenge for these patients. In Fayoum governorate, 20.7% of patients had HCV antibody and 22 out of 25 had PCR confirmation of HCV infection, or 18.2% of all patients [116]. In a patient cohort of 150 patients with a median age of 13 years from Oman, HCV antibodies were detectable in 35.3% [117].

Although the risk of contracting hepatitis C through transfusion is close to zero in the high-income countries, sporadic cases of person-to-person transmission among thalassemia patients are still described. Mazzucco et al. (2020) reported an HCV outbreak among 128 thalassemia outpatients followed at a thalassemia center of an Italian hospital. Combining root cause analysis and molecular epidemiology effectively traced an HCV outbreak to a procedural error during management of peripheral venous access for blood transfusion [118].

## 8. Conclusions

In resource-rich countries, the impact of hepatitis C on patients with thalassemia who were transfused before the advent of the donor screening has been devastating, both epidemiologically and in terms of complications. The real burden of infection emerged when, thanks to oral chelators and non-invasive techniques for preclinical diagnosis of myocardial iron accumulation, patient prognosis improved and they lived longer. The availability of antiviral therapies, only partially effective and causing profoundly impactful side effects on patients’ lives, has meant that in the many patients who have developed chronic infection, the virus has often had the opportunity to exert its damaging effect on the liver and extrahepatically for decades. From this point of view, iron accumulation, secondary to transfusion therapy and increased gut absorption, has been a powerful ally of the C virus, enhancing its negative potential. Only recently has the use of DAAs made it possible to treat the majority of patients with thalassemia and hepatitis C in countries in which DAAs are freely available. However, this achievement, while extremely significant, does not erase the effects of the virus in terms of fibrogenesis and mutagenic risk. As in the general population, it is in fact mainly patients with cirrhosis who are increasing in age, even though they are now HCV RNA-negative, who are at risk of hepatocellular carcinoma, which continues to be statistically much more frequent in individuals with thalassemia than in those without thalassemia. Therefore, even after HCV RNA clearance, regular ultrasound screening is essential in order to detect suspicious lesions early and offer the patient a better chance of cure. Thalassemia per se should no longer be considered a condition that excludes the possibility of liver transplantation, and each patient should be considered individually in a multidisciplinary approach. A subgroup of patients, moreover, have irreversible extrahepatic complications clearly related to the C virus, such as neuropathy and systemic vasculopathy. The role in affecting the severity and prognosis of other organ complications such as heart disease and osteoporosis remains less clear.

In many countries around the world, patients with thalassemia continue to contract hepatitis C, often as children. They often live in realities that do not allow them to become adults, and anemia and heart disease are still the leading causes of death. Increasing the safety of transfusion therapy is therefore one of the priorities alongside the possibility of an adequate transfusion regimen, the availability of the three chelators, and adequate multidisciplinary follow-up. In this context, which should ensure that even those born in the most disadvantaged countries have the same opportunities in terms of open prognosis and quality of life as those who, without merit, were born in wealthier countries, the eradication of hepatitis C in patients who are already infected is clearly essential.

## Figures and Tables

**Table 1:** Patients with beta thalassemia who developed hepatocellular carcinoma after sustained virological response (SVR).

Patients (n°)	Geographic Origin	DAAs as Antiviral Treatment Determining SVR (n° (%))	Cirrhosis (n° (%))	Reference
19	Italy	11 (58)	13/18 (72)	Origa et al., 2023 [34]
4	Italy	2 (50%)	NA	Ricchi et al., 2021 [50]
25	Greece	17 (68)	20 (80)	Papadopoulos et al., 2020 [49]
2	Italy	1 (50)	1 (50)	De Sanctis et al., 2020 [53]
1	Oman	0	1 (100)	
1	Greece	1 (100)	NA	

DAAs: Direct antiviral agents; SVR: Sustained Virological Response; NA: Not Available.
